# Invasive Group B Streptococcal Disease in Neonates and Infants, Italy, Years 2015–2019

**DOI:** 10.3390/microorganisms9122579

**Published:** 2021-12-13

**Authors:** Roberta Creti, Monica Imperi, Alberto Berardi, Erika Lindh, Giovanna Alfarone, Marco Pataracchia, Simona Recchia, The Italian Network on Neonatal and Infant GBS Infections

**Affiliations:** 1 Department of Infectious Diseases, Istituto Superiore di Sanità, 00161 Rome, Italy; monica.imperi@iss.it (M.I.); erika.lindh@helsinki.fi (E.L.); giovanna.alfarone@iss.it (G.A.); marco.pataracchia@iss.it (M.P.); simona.recchia@iss.it (S.R.); 2 Neonatal Intensive Care Unit, Azienda Ospedaliero-Universitaria Policlinico, 41125 Modena, Italy; alberto.berardi@unimore.it; 3 European Centre for Disease Prevention and Control (ECDC), European Program for Public Health Microbiology Training (EUPHEM), SE-171 83 Stockholm, Sweden

**Keywords:** group B streptococci, GBS, *Streptococcus agalactiae*, surveillance, multi-drug resistant CC17 sub-clone, neonatal invasive GBS infection

## Abstract

Invasive infections by group B streptococci (iGBS) are the leading cause of sepsis and meningitis in the first three months of life worldwide. The clinical and microbiological characteristics of neonatal and infant iGBS in Italy during the years 2015–2019 were investigated. Voluntary-based surveillance reported 191 cases (67 early-onset (EOD) and 124 late-onset disease (LOD)) and 89 bacterial isolates were received. The main clinical manifestations were sepsis (59.2%) followed by meningitis (21.5%), bacteremia (12.0%) and septic shock (6.3%). Hospitalized preterm babies accounted for one third of iGBS and constituted the most fragile population in terms of mortality (8.2%) and brain damage (16.4%). GBS serotype III was predominant in EOD (56%) and caused almost all LOD (95%). The rate of resistance to clindamycin reached 28.8%. Most of clindamycin-resistant GBS strains (76%) were serotype III-ST17 and possessed the genetic markers of the emerging multidrug resistant (MDR) CC-17 sub-clone. Our data revealed that iGBS is changing since it is increasingly reported as a healthcare-associated infection (22.6%), mainly caused by MDR-CC17. Continuous monitoring of the clinical and microbiological characteristics of iGBS remains of primary importance and it represents, at present, the most effective tool to support prevention strategies and the research on the developing GBS vaccine.

## 1. Introduction

Group B streptococcus (GBS, *Streptococcus agalactiae*) was first recognised as an emerging pathogen of neonatal invasive bacterial infections in high-income countries fifty years ago. It still remains the most frequent cause of meningitis and a prominent pathogen in sepsis in the first three months of life [1,2]. Conventionally, two forms of the disease are distinguished: one with an early onset (GBS-EOD) from birth through six days of age and one with a late onset (GBS-LOD) within the first three months of life [3,4,5].

Maternal GBS colonization of the genitourinary and gastrointestinal tracts is the main risk factor for EOD. Vertical transmission usually occurs during labor or after rupture of membranes. Implementation of intrapartum antibiotic prophylaxis (IAP) has demonstrated efficacy for prevention of GBS-EOD [4,5,6,7].

Pregnant people eligible to IAP are identified by means of two strategies: (i) the antenatal microbiological screening based on a vaginal-rectal culture at the 36–37th gestational week and (ii) a risk-based approach relying on the evaluation of obstetrical risk factors at delivery: preterm birth (<37 weeks’ gestation), maternal fever ≥ 38 °C, rupture of the amniotic membrane lasting more than 18 h (ROM). GBS bacteriuria during pregnancy or a previous infant with invasive GBS disease are high risk conditions for which the parturient always receives IAP [4,5,8,9].

With implementation of universal maternal antenatal screening and IAP, the incidence of GBS-EOD in the US has declined from 1.8 cases per 1000 live births (with a mortality rate of 25%) in 1990 to 0.23 cases per 1000 live births (with a mortality rate of 7%) in 2015 [8,10]. The incidence rate of GBS-LOD has not been influenced by IAP and fluctuates around 0.3 cases per 1000 live births.

Risk-factors for LOD and route of transmission of GBS are still poorly understood, hindering efforts for prevention [11]. Maternal colonization at the time of LOD diagnosis is frequently detected but additional sources of GBS (from environment and nonmaternal caregivers) are also thought to play a role in the development of the invasive infection [12,13].

Ten immunologically distinct serotypes of GBS have been defined, based on surface polysaccharides (Ia, Ib, II to IX). Serotypes I–V account for about 97% of all serotypes responsible for neonatal infections worldwide [14]. Globally, serotype III strains are clinically the most important, accounting for approximately 25% of colonizing strains and 60% of strains causing invasive disease in neonates and infants, although geographical variation exists [14,15]. In Italy, serotype III accounted for 47.6% of EOD cases, 87.7% of LOD cases and 38.3% of maternal colonizing strains in the years 2007–2014 [16]. The serotype distribution of GBS strains isolated from both EOD and maternal colonization was comparable confirming the neonatal bacterial acquisition by vertical transmission. On the contrary, serotype III predominated in GBS-LOD [16,17].

In Italy, late third-trimester antenatal screening and IAP are recommended to lower the risk of GBS-EOD [18]. There are not national data on the incidence of neonatal invasive GBS disease; nevertheless, regional area-based studies have reported a sharp decline in the incidence of EOD after the implementation of targeted intravenous IAP [19,20,21,22,23].

The National Reference Laboratory at the Istituto Superiore di Sanità (ISS-NRL) for neonatal GBS disease maintains an epidemiological and microbiological surveillance.

Data obtained in the years 2015–2019 were analyzed in order to understand: (i) the evolving characteristics of neonatal and infant invasive GBS disease (iGBS), (ii) to evaluate missed opportunities for prevention and possible corrective actions, (iii) the fluctuation of serotypes responsible for iGBS and their antimicrobial susceptibility. This information is essential to predict at what extent the introduction of a GBS vaccine will mitigate the burden of iGBS.

## 2. Materials and Methods

### 2.1. Data Collection

Cases of invasive GBS neonatal disease were reported to ISS-NRL on a voluntary basis by 26 hospitals across the country and by the active area-based surveillance network of Emilia Romagna. In particular, the collaborating 26 hospitals were located in Northern Italy (eleven birth centers), Central Italy (eleven birth centers), Southern Italy (four birth centers). The network of Emilia Romagna (Northern Italy) is composed by 18 hospitals and was established since the year 2003 [24].

A questionnaire, tailored for the iGBS surveillance, included both anonymous maternal data (antenatal screening, gestational age at childbirth, mode of delivery, risk factors and IAP administration) and neonatal data (sex, ethnicity, onset of symptoms, clinical manifestation, outcome).

Inclusion criteria were only culture-proven cases from a normally sterile site (blood and/or cerebrospinal fluid) in babies aged 0–90 days, reported from 1 January 2015 to 31 December 2019.

Exclusion criteria were clinical sepsis, stillbirths, iGBS over 90 days of life.

### 2.2. Bacterial Collection Typing

When GBS isolates were sent to ISS-NRL, species confirmation was done by the determination of group B Lancefield surface antigen using the Streptococcal grouping kit (Oxoid). Serotyping was based on commercial latex agglutination test ImmuLexTM StrepB-Kit (SSI Diagnostica, Hillerød, Denmark) [25,26]. Molecular typing of capsular types Ia-IX was performed using a multiplex-Polymerase Chain Reaction (PCR) assay both in case of not-typeable strains and for confirming the results of the agglutination test [27]. Antimicrobial resistance profile to erythromycin, clindamycin and tetracycline was performed as already described [17].

Identification of hypervirulent ST-17 lineage was performed by using a PCR assay based on the detection of the *hvg*A gene [28]. Pilus island gene content was performed by a PCR assay that identifies the presence of pilus island (PI) 1, PI-2a and PI-2b [29].

### 2.3. Statistical Analysis

Data were analyzed using STATA/SE 14.2 for Windows and MedCalc version 9.3 (MedCalc Software, Ostend, Belgium). Continuous data that were non-normally distributed were described as medians with interquartile ranges (IQRs); categorical data were described as numbers and percentages. Student’s t test or the Mann-Whitney U test and x2 test or Fisher’s exact test were used to compare continuous and categorical variables between groups. Two-sided *p* values < 0.05 were considered significant.

## 3. Results

### 3.1. Characteristics of iGBS

In the years 2015–2019, the ISS-NRL received 191 reports of iGBS (67 EOD and 124 LOD). Of these, 47.6% cases (*n* = 91) came from the active surveillance network of Emilia Romagna which reported 37.3% (*n* = 25) and 53.2% (*n* = 66) of EOD and LOD, respectively.

Neonatal median age in EOD was 4 h but most newborns (82.0%, *n* = 55) developed symptoms within the first day of life. Asymptomatic bacteremia was associated to EOD; conversely, meningitis and prematurity was associated to LOD (Table 1). The prolonged hospital stays (median 36.5 days) of pre-term newborns along with their immature immune system could have increased the risk for developing LOD and for the observed higher mortality and brain damage than term neonates (Table 2).

During the study period, two overlapping and distinct clusters of GBS-LOD affected early preterm infants admitted to the same neonatal intensive care units (NICU) [30]. Overall, twenty-eight hospitalized preterm infants presented with GBS-LOD (22.6%) of which five presented a recurrence by the same GBS strain.

### 3.2. Prevention Strategies and Missed Opportunities in GBS-EOD

Thirty-two newborns who developed EOD were delivered to mothers with positive GBS screening or with risk factors. IAP was administered in only ten cases and always as an incomplete course (less than 4 h duration) (Table 3).

Ten parturients presented with a fever at delivery and a negative GBS colonization status; regardless of whether they received an antibiotic treatment, all their full-term babies were already septic at birth or after few hours.

It was worrying to note that 23 newborns (34.3%) developed EOD despite the absence of any indication for IAP administration because no risk factors were present at delivery and the GBS colonization maternal status was negative (20 cases) or unknown (three cases) (Table 3).

### 3.3. Bacterial Typing and Antimicrobial Resistance

**Figure 1:**
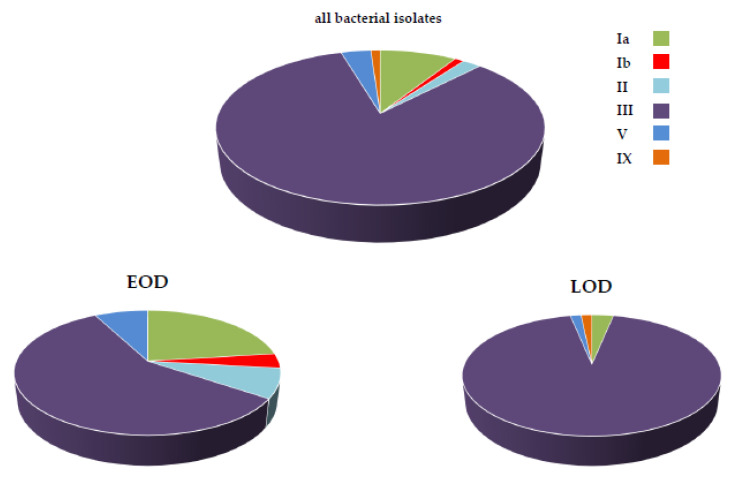
Distribution of GBS serotypes responsible for iGBS.

About half (46.6%) of neonatal iGBS disease reports were accompanied by the bacterial shipping to ISS-NRL (89 strains). The most frequent serotype was type III (74 strains, 83.4%), followed by Ia (8 strains, 9.0%), V (3 strains, 3.4%), II (2 strains, 2.2%), Ib and IX (one strain each, 1.1%) (Figure 1). Of these, 11.2% of the bacterial strains did not express surface polysaccharides and could be serotyped only using the multiplex-PCR assay. Remarkable differences in the distribution of serotypes responsible for EOD and LOD were observed. While serotype III accounted for about half of the EOD cases (57.7%, *n* = 15), it could be isolated from nearly all (93.6%, *n* = 59) LOD cases (Figure 1). Most serotype III strains isolated both from EOD (86.7%, *n* = 13) and LOD (89.8%, *n* = 53) were classified as ST-17 by the specific PCR assay.

The constitutive resistance to both clindamycin and erythromycin (mediated by the *erm*B gene) was expressed by 25.8% (*n* = 23) GBS strains; inducible resistance to clindamycin (mediated by the *erm*A gene) was expressed by two strains. No antimicrobial resistance mediated by the efflux pumps genes *mef*A/E was identified (Table 4).

Antimicrobial resistance to tetracycline was possessed by 92.1% GBS strains; *tet*M gene was the most diffuse genetic determinant (73.2%), associated to erythromycin resistance only in two EOD cases (the *erm*A strains). Conversely, *tet*O gene was always associated with *erm*B gene, except for one case (Table 4). The *tet*O-*erm*B genes association was possessed by GBS serotype III-ST17 strains that lacked the pilus island (PI) 1. This feature (presence of *erm*B-*tet*O genes and absence of PI-1) is strongly suggestive of the emerging Multi Drug Resistant (MDR) hypervirulent clonal complex (CC)-17 sub-lineage, worldwide reported [31,32,33].

## 4. Discussion

Our study defined the clinical and microbiological characteristics of iGBS disease in neonates and infants younger than 90 days in Italy in years 2015–2019. While consistent with microbiological and epidemiological data on iGBS from other developed countries, this study provided new and updated information on risk factors, disease presentation and circulating serotypes. In particular, we have described the increased reporting of LOD in NICU among pre-term infants and of a multidrug-resistant GBS CC17-subclone.

### 4.1. Prevention

Public health recommendations for the prevention GBS-EOD were launched 25 years ago. Nevertheless, GBS continues to be one of the most frequent organism causing early-onset sepsis in both developing and developed countries [6,34,35,36]. In the Netherlands and the UK, the incidence of GBS-EOD is even increased [37,38] despite the introduction of national prevention guidelines. This has given rise to concern and a request to review the strategies adopted.

The present study indicates that further efforts in the prevention of GBS-EOD are likewise desirable in Italy. Our data indicate that 44.7% (*n* = 33) of EOD cases had mothers who tested negative to the antenatal cultural screening for GBS, therefore with no indication for the administration of IAP. Similarly, a multistate and population-based surveillance in the US during the years 2006–2015 reported that 40% EOD cases were attributable to an antenatal screening negative for GBS [39]. Since GBS-EOD results from the vertical transmission of GBS from colonized mother to the newborn, it is likely that the GBS test result had, at least for a part of the cases, an inadequate sensitivity. When laboratory testing for GBS is performed on schedule (at 36–37 weeks’ gestation), using the correct sampling method (collection at both vaginal and rectal site) and following the recommended procedure (pre-enrichment in broth and then plating on selective medium) the result is extremely reliable [8,40,41]. A pre-enrichment step is strongly recommended also in the case of the use of Nucleic Acid Amplification Test (NAATS) [40].

Unfortunately, not all centers provided information on the microbiological procedure for the detection and identification of GBS at prenatal screening and therefore it was not possible to ascertain the true proportion of appropriately performed GBS tests. A national survey published a few years ago had indeed suggested that maximizing adherence to recommended microbiological practices was desirable to further reduce the burden of EOD. The survey reported that only 62.8% of national clinical microbiology laboratories collected both a vaginal and rectal swab and 58% of them performed a pre-enrichment step [42].

Another important missed opportunity for prevention was not to administer IAP despite indications to do so. In our study, 100 women were eligible for IAP administration at delivery, but only 51 received at least one antibiotic dose [9]. Unfortunately, this represents the most difficult aspect to ameliorate given the limited information available. Precipitous delivery was cited as the cause of missed IAP in about 30% of cases but, in the remaining cases, no obvious reason for missed prevention could be evinced.

### 4.2. Clinical Epidemiology and Risk Factors

Our data indicate that clinical presentation differed according to age at onset: GBS-EOD presented more frequently with asymptomatic bacteremia and GBS-LOD more likely presented with meningitis. Although iGBS affected more term than preterm neonates, those with a low gestational age had a higher risk of mortality and brain damage. Furthermore, prematurity was significantly associated to GBS-LOD, presenting an increasing trend compared to our previous reporting period [17]. Our findings are consistent with other studies [39,43]. A large continuous study comprising more than fifteen thousand infants for twenty years in UK highlighted that low gestational age at birth was associated with 5.2 to 22-times higher odds of GBS diagnosis [44]. A very comprehensive Swedish retrospective study that encompassed forty-three years of surveillance on LOD (using the case definition of symptoms from 72 h of life onward) demonstrated that GBS-LOD incidence significantly doubled in the last 20 years (from 0.16 to 0.33/1000 livebirths). The increase of infection mostly regarded infants born extremely preterm and it represented the first cause of meningitis [45].

The greater instrumental and caring capacity is able, nowadays, to support early-preterm neonates but, due to the prolonged hospital stay, their risk of acquiring healthcare associated infections has increased. In our study, nosocomial GBS-LOD cases accounted for almost one quarter of all late-onset infections; five infants re-presented with GBS infection while still in NICU. A recent systematic review to characterize outbreaks of invasive GBS disease in hospitals focused on 30 clusters (26 neonatal, 4 adult) in 11 countries from 1966 to 2019, demonstrating how these events mostly occurred during periods of crowding and high patient-to-nurse ratio [46]. An in depth-analysis by using whole genome sequencing of invasive GBS isolates collected during a 13 month-period of enhanced national surveillance of infant invasive GBS disease in the UK and Ireland (years 2014–2015) reported that approximately one in twelve LOD cases were part of a hospital cluster. Over half of clusters were previously undetected, emphasizing the importance of routine submission of GBS isolates to reference microbiology laboratories for their rapid identification and reporting [47]. A fine molecular typing of GBS isolates during the nosocomial outbreak of GBS-LOD that occurred in this five-year study period indicated that it was caused by two independent and overlapping introductions of GBS [30].

The long-term follow-up of iGBS is an extremely important and underestimated topic that deserves further consideration. In our study, 8.4% of infants who recovered from iGBS had brain lesions at hospital discharge. A national matched cohort study that was conducted in Netherlands and Denmark to assess long-term mortality, neurodevelopmental impairments and economic outcomes after iGBS through adolescence, pointed out that GBS meningitis was associated with increased mortality at age 5 and iGBS was associated with an increased risk of neurodevelopmental impairments at 10 years of age [48].

### 4.3. Microbiological Epidemiology, Antibiotic Resistance and the Emergence of MDR CC-17 Sub-Lineage

The present study confirmed the predominance of serotype III as responsible for neonatal and infant iGBS: it caused 57.7% of GBS-EOD but reached an incidence of 93.6% in LOD.

The pivotal role of serotype III in neonatal GBS invasive disease is mainly due to the success of a single clonal lineage, defined as Clonal Complex 17 (CC-17) and diffused worldwide [6,31,43,49,50]. The reason for the monoclonal nature of GBS in the aetiology of LOD has been only partially disentangled: the meningeal tropism of GBS serotype III-CC17 is mediated by exclusive surface adhesins [51] and the fine-tuning of both transcription and expression of virulence factors has been postulated to modulate the transition from the carrier state to pathogenicity [52,53].

The successful fitness GBS serotype III CC-17 is gaining much attention with recent reports on the emergence of a multidrug resistant (MDR) CC-17 sub-lineage [31,32,33,54]. The GBS type III MDR CC-17 sub-clone possesses integrative and conjugative elements (ICE) conferring additional resistance to tetracycline, macrolides and lincosamides and exhibiting high-rate resistance to aminoglycosides. Genomic studies demonstrated that the ICE elements replace the 16-Kb genome region that comprises the entire pathogenicity island encoding the pilus 1 [31,32]. First reported from the analysis of genomic GBS CC-17 strains isolated from neonatal infections in Southern China in the years 2013–2014 [31], retrospective studies have indeed demonstrated that this MDR CC-17 sub-clone circulated in Canada, Portugal and France since the year 2010 [32,33,54]. Even if we did not perform a detailed genomic analysis of all GBS strains, we can confidently assume that the MDR CC-17 sub-clone was responsible for the observed alarming increase in both erythromycin and clindamycin resistance that was doubled respect to the past surveillance (15% in the years 2007–2014 [17] and almost 30% in this five years’ period). The hospital outbreak that occurred during the study period was caused by the overlapping introduction of two different GBS serotype III-MDR-CC17 clones [30]. Reports on the increasing rate of clindamycin resistance in GBS are always more frequent and clindamycin resistant GBS has been included in the CDC’s 2019 Antibiotic Resistance Threats Report.

The high-level aminoglycoside resistance and reports on reduced susceptibility to beta-lactams [55] indicate that continuous monitoring and deep genetic analysis is desirable to timely capture possible clonal expansion of MDR-GBS and to enhance infection control programs [55]. A low frequency of the MDR ICE elements in other clonal lineages (ST-12 and ST-23 genomes) has also been described [31,55]. In our study, two non-ST-17 strains (one serotype Ib and one serotype II) lacked pilus island-1 and additionally harboured the *erm*B and *tet*O genes. Then, even if these resistant genotypes have not expanded in a manner similar to MDR-CC17, nevertheless they should deserve to be closely monitored. Overall, 24.7% (*n* = 22) iGBS cases in this study were indeed caused by a MDR clone. In particular, it was responsible for 15.4% and 25.4% of EOD and LOD cases, respectively. This urges a particular attention in settings such as in neonatal intensive care units where extensive antibiotic use and prolonged hospital stay are common. Here, multidrug resistance constitutes a relevant concern for potential therapeutic failures or reduced synergistic action of the beta lactam-aminoglycoside regimen that is used as empiric therapy in neonatal sepsis.

### 4.4. GBS Vaccine

The present data confirm the effectiveness of IAP, although residual cases of GBS-EOD continue to occur. While in our past study EOD and LOD cases were numerically equivalent [17], in this five-year surveillance LOD exceeded EOD by almost two-fold. The predominance of GBS-LOD is reported by other high-income countries [39,50]. This means that more than half of the neonatal GBS infections now occur in a form that is no longer preventable. With GBS causing 147,000 stillbirths and infant deaths annually, development of GBS vaccines for maternal immunization has been identified as a priority for the WHO Initiative for Vaccine Research [1]. More advanced vaccines are constituted by glycoconiugates comprising the most diffuse capsular polysaccharide types but other formulations that are composed by only GBS protein antigens are in development [15]. The hexavalent GBS glycoconiugate vaccine that actually satisfied PhaseI/II clinical trials encompasses the serotypes Ia, Ib, II, III, IV, V. Our data confirmed that it will provide excellent coverage against the vast majority of iGBS cases in Italy, reducing iGBS-associated morbidity and deaths as well as the antibiotic use [56,57,58].

### 4.5. Limitations

Some important limitations of our study should be acknowledged. First, our data are based on a voluntary surveillance. Although cases were reported across the country, they may not reflect the national situation. Capillary coverage of population is difficult to obtain even with active surveillance. For example, the CDC Active Bacterial Core surveillance includes approximately 445,000 live births annually, corresponding to 12.4% of total births.

The cases reported by the Emilia-Romagna network represented almost half of the total Italian cases, indicating a substantial underreporting that may have affected the real proportion of GBS serotypes (i.e., the predominance of serotype III) and the rate of antibiotic resistance.

Finally, we do not know the number of very preterm births in each center, therefore we cannot precisely assess their risk of iGBS, although previous Italian regional area-based studies have provided this information [12,21].

## 5. Conclusions

Our study revealed that iGBS is changing: post-natal bacterial exposure and horizontal transfer now constitute the main risk factors for iGBS disease. At present, iGBS is more diffused as late onset disease and increasingly reported as a healthcare associated infection among pre-term infants in NICU.

Antibiotic resistance rate, in particular clindamycin resistance, has doubled compared to the previous surveillance period due to the increased diffusion of the serotype III MDR-CC17 subclone. These findings provide a strong rationale for the continuous monitoring of the clinical and microbiological characteristics of iGBS in Italy.

## Figures and Tables

**Table 1:** Demographic and clinical characteristics of iGBS.

	EOD *n* = 67 (35%)	LOD *n* = 124 (65%)	All Cases (*n* = 191)
**Males, n (%)**	32 (47.8)	63 (50.8)	95 (49.7)
**Gestational age at delivery, median, week (IQ)**	39 (37.7–40)	38 (33–39)	38 (34.8–40)
**Age at onset of disease, median, (IQ)**	4 h (0–18.5)	30 days (17.5–44)	not done
**Ethnicity n (%)**			
African	5 (7.5)	16 (12.9)	21 (11.0)
Arab	1 (1.5)	4 (3.2)	5 (2.6)
Asian	3 (4.5)	3 (2.4)	6 (3.1)
White	58 (86.6)	101 (81.4)	159 (83.3)
**Clinical diagnosis, n (%)**			
Sepsis	35 (52.2)	78 (62.9)	113 (59.2)
Asymptomatic bacteremia	**21 (31.3)**	2 (1.6)	23 (12.0)
Meningitis	9 (13.4)	**32 (25.8)**	41 (21.5)
Septic shock	2 (3.0)	10 (8.0)	12 (6.3)
Arthritis, osteomyelitis	0	2 (1.6)	2 (1.0)
**Bacterial isolation, n (%)**			
Blood	60 (89.5)	89 (71.8)	149 (78.0)
Cerebrospinal fluid	2 (3.0)	2 (1.6)	4 (2.1)
Blood and cerebrospinal fluid	5 (7.5)	33 (26.6)	38 (19.9)
**Outcome, n (%)**			
Full recovery	57 (85.1)	108 (87.1)	165 (86.4)
Brain lesions	4 (6.0)	12 (9.7)	16 (8.4)
Deceased	6 (8.9)	4 (3.2)	10 (5.2)
**Risk factors n (%)**			
None	37 (55.2)	64 (51.6)	101 (52.9)
1	21 (31.3)	49 (39.5)	70 (36.6)
>1	7 (10.4)	5 (4.0)	12 (6.3)
Not reported	2 (3.0)	6 (4.8)	8 (4.2)
**Risk factors, specified, n (%)**			
Prematurity (<37 weeks)	12 (17.9)	**49 (39.5)**	61 (31.9)
Intrapartum fever ≥ 38 °C	12 (17.9)	1 (0.8)	13 (6.8)
Bacteriuria	4 (6.0)	2 (1.6)	6 (3.1)
Amniotic membrane rupture > 18h	8 (11.9)	7 (5.6)	15 (7.8)
GBS antenatal screening, n (%) *			
Not done	4/53 (7.5)	9/74 (12.1)	13/127 (10.2)
Not reported	2/53 (3.8)	5/74 (6.7)	7/127 (5.5)
Negative	28/47 (59.6)	30/60 (50.0)	58/107 (54.2)
Positive	19/47 (40.4)	30/60 (50.0)	49/107 (45.8)
**Mode of delivery, n (%)**			
Vaginal	44 (65.7)	67 (54.0)	111 (58.1)
Planned caesarean section	1 (1.5)	26 (21.0)	27 (14.1)
Emergency caesarean section	20 (29.8)	24 (19.3)	44 (23.0)
Not reported	2 (3.0)	7 (5.6)	9 (4.7)

Numbers and percentages in bold are statistically significant (*p* < 0.05) * only antenatal microbiological screening performed according current recommendations (after 36th week of gestation) has been considered.

**Table 2:** Hospital stay, mode of delivery and outcome in pre-term newborns and full-term newborns *.

	Pre-Term Infants (*n* = 61, 31.9%)	Term Infants (*n* = 119, 62.3%)	*p* Value
**Hospital stay, median, days (IQ)**	36.5 (14.7–64.5)	12 (10–16)	
**Mode of delivery, n (%)**			
vaginal	19 (31.1%)	90 (75.6%)	<0.0001
planned caesarean section	14 (23.0%)	12 (10.1%)	0.04
emergency caesarean section	28 (45.9%)	14 (11.8%)	<0.0001
not available	--	3	
**Outcome**			
death	5 (8.2%)	3 (2.5%)	0.12
brain lesions at discharge from hospital	10 (16.4%)	6 (5.0%)	0.02

* information on gestational age was not available for 11 cases.

**Table 3:** Policies adopted at delivery in GBS-EOD *.

	IAP Administrated	IAP Not Administrated
any indication for IAP	10	22
(32 cases)		emergency CS (8 cases)
		unknown reason (14 cases)
		
no indication for IAP	7	26
(33 cases)	(maternal GBS negative status but fever) **	unknown maternal GBS status and no risk factors (3 cases)
		maternal GBS negative status and no risk factors (20 cases)
		maternal GBS negative status but fever (3 cases)

* information was missing in two cases ** ACOG 2020 recommendations state that IAP is not indicated in case of “Negative vaginal-rectal GBS culture obtained at 36 0/7 weeks of gestation or more during the current pregnancy, regardless of intrapartum risk factors” IAP: intrapartum antibiotic prophylaxis CS: caesarean section.

**Table 4:** Description of erythromycin and tetracycline resistant GBS strains.

	Ery Res Strains (Number, %)	Serotypes	ST-17	MDR* ST17 (Number, %)	Erythromycin Resistance Genes (Number, %)	Tet Res Strains (Number, %)	Tetracycline Resistant Genes (Number, %)
**EOD**	8 (30.8%)	Ib (1), II (1), III (6)	6	4 (66.7%)	*erm*B (6, 75%); *erm*A (2, 25%)	25 (96.1%)	*tet*M (21; 80.8%); *tet*O (6; 23.1%)
**(26)**							
**LOD**	17 (27.0%)	III (17)	17	15 (88.2%)	*erm*B (17, 100%)	57 (90.5%)	*tet*M (44, 69.8%); *tet*O (16, 25.4%);
**(63)**							*tet*M + *tet*O (1; 1.6%)
							
**Total**	25 (28.1%)		23 (92%)	19 (76%)	*erm*B (23, 92%); *erm*A (2, 8%)	82 (92.1%)	*tet*M (66, 74.1%); *tet*O (23, 25.8%)

* presence of the genes *ermB*, *tet*O and lack of PI-1.

## Data Availability

Not applicable.
